# Pathology and Molecular Epidemiology of Fowl Adenovirus Serotype 4 Outbreaks in Broiler Chicken in Abu Dhabi Emirate, UAE

**DOI:** 10.3390/vetsci9040154

**Published:** 2022-03-23

**Authors:** Hassan Zackaria Ali Ishag, Abdelnasir Mohammed Adam Terab, El Tigani Ahmed El Tigani-Asil, Oum Keltoum Bensalah, Nasereldien Altaib Hussein Khalil, Abdelmalik Ibrahim Khalafalla, Zulaikha Mohamed Abdel Hameed Al Hammadi, Asma Abdi Mohamed Shah, Salama Suhail Mohammed Al Muhairi

**Affiliations:** Veterinary Laboratories Division, Animal Wealth Sector, Abu Dhabi Agriculture and Food Safety Authority (ADAFSA), Abu Dhabi P.O. Box 52150, United Arab Emirates; abdelnasir.terab@adafsa.gov.ae (A.M.A.T.); eltigani.mohammed@adafsa.gov.ae (E.T.A.E.T.-A.); oumkeltoum.bensalah@adafsa.gov.ae (O.K.B.); nasareldien.khalil@adafsa.gov.ae (N.A.H.K.); abdelmalik.khalafalla@adafsa.gov.ae (A.I.K.); zulaikha.alhammadi@adafsa.gov.ae (Z.M.A.H.A.H.); asma.mohammed@adafsa.gov.ae (A.A.M.S.); salama.almuhairi@adafsa.gov.ae (S.S.M.A.M.)

**Keywords:** fowl adenoviruses, inclusion body hepatitis, hydropericardium syndrome, epidemiology, UAE

## Abstract

Background: Fowl adenovirus serotype 4 (FAdV-4), causing inclusion body hepatitis (IBH) and hydropericardium hepatitis syndrome (HPS), is responsible for the significant economic losses in poultry industry worldwide. This study describes FAdV disease and molecular characteristics of the virus as the first report in UAE. Methodology: Clinical, necropsy, histopathology, qPCR and phylogenetic analysis of hexon gene were used to diagnose and characterize the virus. Results: The age of the infected broiler chicken was 2–4 weeks. The morbidity and mortality rates ranged between 50 and 100% and 44 and 100%, respectively. Clinically, sudden onset, diarrhea, anemia and general weakness were recorded. At necropsy, acute necrotic hepatitis, with swollen, yellowish discoloration, enlarged and friable liver; hydropericarditis with hydropericardium effusions; and enlarged mottled spleen were observed. Histopathology examination revealed degeneration and necrosis, lymphocytic infiltration and inclusion bodies. The qPCR analysis detected the virus in all samples tested. Hexon gene sequence analysis identified FAdV serotype 4, species C as the major cause of FAdV infections in UAE in 2020, and this strain was closely related to FAdV-4 circulating in Saudi Arabia, Pakistan, Nepal and China. Conclusion: The serotype 4, species C, was the common FAdV strain causing IBH and HPS episodes in the region. This result may help design effective vaccination programs that rely on field serotypes.

## 1. Introduction

Adenoviruses infection is one of the most prevalent diseases in a wide range of poultry species worldwide, resulting in severe economic losses in the poultry industry [1,2]. Fowl adenovirus (FAdV) is a DNA virus belonging to group-I adenoviruses in the genus Aviadenovirus in the family of Adenoviridae [3]. The virus is genetically grouped into five species (FAdV A, B, C, D and E) based on restriction fragment length polymorphism (RFLP) of the full FAdV genome and further classified into twelve serotypes (FAdV-1 to 8a and 8b to 11) by cross-neutralization test [4].

Some of the FAdVs are implicated in a variety of diseases in chicken, including inclusion body hepatitis (IBH), hydropericardium hepatitis syndrome (HPS) and adenoviral gizzard erosion (AGE) [5,6,7]. Both IBH and HPS in broiler chickens could be caused by any of the twelve serotypes of FAdV with a 10–30% mortality rate [8], while HPS is associated with species C (FAdV-C), serotype 4 fowl adenovirus (FAdV-4) with a mortality rate 30–80% [9,10,11]. Moreover, IBH is usually associated with FAdV-2-11 (species D), 8a and 8b (species E), while AGE is commonly caused by FAdV-1 (species A) [12,13,14].

Clinically, the affected chickens by IBH and HPS show depression, ruffled feathers and resting on the ground with their chest close to death [15].

The pathological changes in IBH and HPS adenovirus infection include pale, enlarged, hemorrhagic and friable liver; swollen and hemorrhagic kidneys; and mottled spleen [16]. The most consistent pathological lesions of adenovirus infection observed in broilers were hepatitis with severe hepatomegaly and cholestasis, splenomegaly, hemorrhagic nephritis, hydropericarditis, pancreatitis, tracheitis and proventriculitis, visceral gout and serosal ecchymoses of the gizzard wall [17,18].

Severe microscopic lesions could be observed during the histopathological examination of IBH and HPS infection in several organs [19]. In the liver, multifocal areas of coagulative necrosis and many hepatocytes with large, round, eosinophilic or basophilic intranuclear inclusion bodies were described. In the heart, accumulation of mononuclear cells was observed. There was massive degeneration or necrosis of the acinar epithelium in the pancreas, and viral inclusion bodies appeared in the cell nuclei. In the kidneys, there was severe hyperemia and massive degeneration of the epithelium in all renal tubules. In the lungs, congestion and various degrees of edema were reported. In the brain, there was slight viral encephalitis with lymphocyte infiltration around the blood vessels [19].

Confirmation of FAdV (A-E) diagnosis could be performed by qPCR [20], and further characterization of serotypes and species levels could be performed by direct sequencing of the hexon gene [21]. The hexon protein is a major antigenic determinant and a reliable marker for detailed epidemiological and phylogenetic analyses of FAdV field strains.

The FAdV infection has been described in several Asian and Middle East countries, including Saudi Arabia [22], Iraq [23], Pakistan [24,25], Korea [6], China [10], Japan [7] and India [26]. Most of these FAdV belong to FAdV-4, species C, a new dominant serotype worldwide. In UAE, only a single report describing FAdV infection in Pigeon is publically available [27].

Several breeder farms in the Al Ain region, Abu Dhabi Emirate, UAE, reported high morbidity and mortality rates in broiler chickens aged 2–4 weeks in 2020. Chickens in these farms showed clinical signs and pathological lesions suggestive of FAdV infection. All diagnostic results in these farms confirmed the typical IBH and HPS infection of FAdV in broiler chicken. Therefore, the main goal of the present study is to describe the clinical, pathological features and molecular characteristics of FAdV in broiler chicken based on routine diagnosis in Abu Dhabi Agriculture and Food Safety Authority (ADAFSA) Veterinary Laboratories, Abu Dhabi, UAE. Based on the sequencing of the partial hexon gene, we identified the FAdV serotype and species responsible for the IBH and HPS outbreaks in the region in 2020 and further described the phylogenetic and evolutionary relationships of the predominant FAdV serotype with other FAdV worldwide available in the GenBank. The study’s findings will help understand the epidemiology of FAdV infection and design control measures in the region, especially vaccination programs.

## 2. Materials and Methods

### 2.1. Epidemiological Data, Necropsy and Sampling

**Figure 1:**
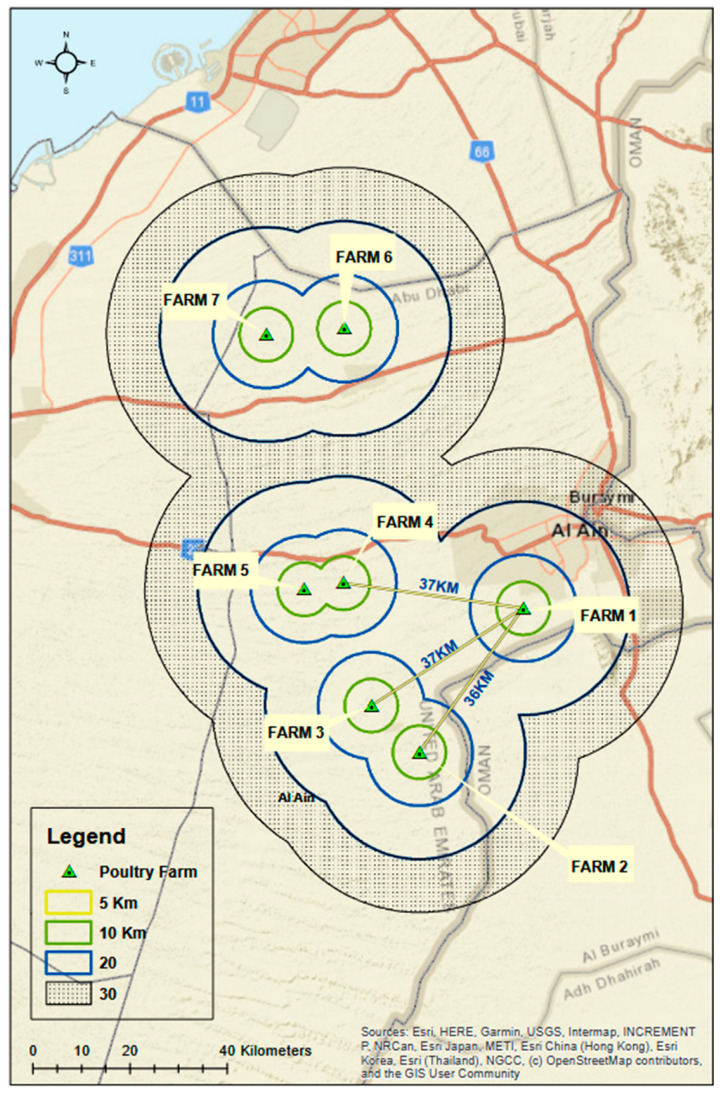
Map showing the location of the seven FAdV infected farms in Al Ain region, Abu Dhabi, UAE. The distance between the infected farms were shown in kilometers.

Between March and December 2020, several FAdV epidemic waves were reported in seven broiler farms in the Al Ain region, Abu Dhabi Emirate, UAE. The location of the farms is shown in (Figure 1). The poultry population per farm ranged from 9000 to 107,839 chickens. The case history, epidemiological data and clinical signs were recorded by ADAFSA veterinarians, and ninety-seven broiler chickens in different ages and stages of the disease and recently dead birds were submitted for routine necropsy examinations. Following necropsy examinations, fifty-four representative tissue samples from different birds, including liver, spleen and heart, were collected and transported on ice to ADAFSA molecular biology laboratory for FAdV PCR confirmation and subsequent analysis. For histopathological examinations, the samples were fixed in 10% neutral formalin. The post-mortem and avian diagnostic pathology were performed following the previously described procedure [28]. All tests performed in this study were in the context of routine diagnosis and clinical activity, and no experimental treatments or additional assays were applied during the study.

### 2.2. Histopathological Examination

Tissue samples, including liver, heart, spleen and kidney, were fixed in 10% neutral formalin for 24–48 h at room temperature for histopathological examinations following the previously described method [29]. The formalin-fixed tissue samples were processed in an automatic tissue processor (ATP1-220, Triangle Biomedical Sciences, Durham, NC, USA), embedded in paraffin blocks and cut into five µm thick sections. Sections were stained with Hematoxylin and Eosin (H & E) (Thermo Fisher Scientific, Runcorn, Cheshire, UK) for microscopic examination, and the images were acquired with the VisionTek digital microscopy system (DM01, Sakura Finetek, Torrance, CA, USA).

### 2.3. SYBR Green-Based Quantitative Real-Time PCR (qPCR) of FAdV

According to procedures previously described, the presence of FAdV in each sample was confirmed by quantitative real-time polymerase chain reaction (qPCR) [20]. DNA extraction was performed using the collected fifty-four tissues samples including liver, spleen, kidney and heart) using the EZ1 DNA Tissue Kit (Qiagen, Hilden, Germany) as per the kit instructions. Briefly, tissue samples were lysed in 300 µL of buffer G2 at 56 °C for 15 min. About 200 µL of the lysate was transferred to the Advanced EZ1 instrument, and the DNA was eluted in 50 µL. DNA quality was measured on a Nanodrop 2000 spectrophotometer (Thermo Scientific, Waltham, MA, USA) and was used either for SYBR Green real-time PCR analysis or subsequent sequencing.

### 2.4. Hexon Gene Sequencing and Phylogenetic Analysis

#### 2.4.1. Hexon Gene Amplification

The Hexon gene (800 bp) was amplified from the extracted DNA of representative samples using AmpliTaq Gold^®^ 360 (Applied Biosystems, Waltham, MA, USA) and a set of previously described Hexon gene primers: HexF1 (5′-GAYRGYHGGRTNBTGGAYATGGG-3′) and HexR1 (5′-TACTTATCNACRGCYTGRTTCCA-3′) [21,30]. These primers were previously designed from a conserved sequence of hexon genes of several FAdVs (group I-III) that can determine the species and serotype of FAdV [21]. The PCR thermal profile was set to 95 °C for 10 min for initial denaturation and activation of the polymerase followed by 35 cycles of 94 °C for 30 s, 55 °C for 30 s and 72 °C for 1 min. The final extension was conducted at 72 °C for 5 min. The amplicons were visualized in a 1.5% agarose gel.

#### 2.4.2. Sanger Sequencing

The PCR products were purified with a QIAquick PCR Purification Kit (Qiagen, Hilden, Germany) and subjected to bidirectional Sanger sequencing using the original Hexon gene primers and BigDye Terminator v3.1 Cycle Sequencing kit (Applied Biosystems) at ADAFSA molecular laboratory. The 10 μL reactions consist of (2.5 µL of DNA, 4 µL of the BigDye Terminator V3.1, 3 µL of water and 0.5 µL of 3.2 pmol primers). The reaction mixture was purified with the BigDye XTerminator™ Purification kit (Applied Biosystems) following the manufacturer’s instructions. Sequencing was performed on a SeqStudio Genetic Analyzer (Applied Biosystems) using the ‘LongSeq BDX’ run module. The sequence trimming and assembly were performed with CLC Genomic Workbench v.20 (Qiagen, Aarhus, Denmark), and the obtained consensus sequence (800 bp) was first subjected to serotype and species identification using the BLAST search tool [31].

#### 2.4.3. Sequence Alignment and Phylogenetic Analysis

For FAdV serotyping, comparative sequence analysis of the partial hexon gene obtained in this study along with forty-one reference sequences of corresponding hexon genes representing all the groups and serotypes of FAdV were used in the phylogenetic tree as indicated previously [6]. These sequences were either obtained from the NCBI nucleotide sequence database or from previously published papers [19]. Multiple sequences alignment was performed with the ClustalW program [32] impeded in MEGAX. The phylogenetic tree was built with the Maximum Likelihood method and Kimura 2-parameter model [33] with 1000 Bootstrap confidence using the MEGAX software [34].

## 3. Results

### 3.1. Epidemiological Data and Sampling

Nine epidemic waves of IBH or HPS were recorded in seven broilers chicken farms located in the Al Ain region, Eastern Abu Dhabi, from March to December 2020. The infected farms were at least 10 Kilometers distant from each other (Figure 1). The poultry population per farm was found to be ranging from 9000 to 107,839 chickens. The birds showed high morbidity, mortality and case-fatality rates on the date of notification ranging between 50 and 100%, 44 and 100%, and 67 and 100%, respectively (Table 1). The age of the infected chickens was between 2 and 4 weeks. The mortality rate is negatively correlated with age as it increases to 100% in small age broilers.

### 3.2. Clinical and Pathological: Findings

**Figure 2:**
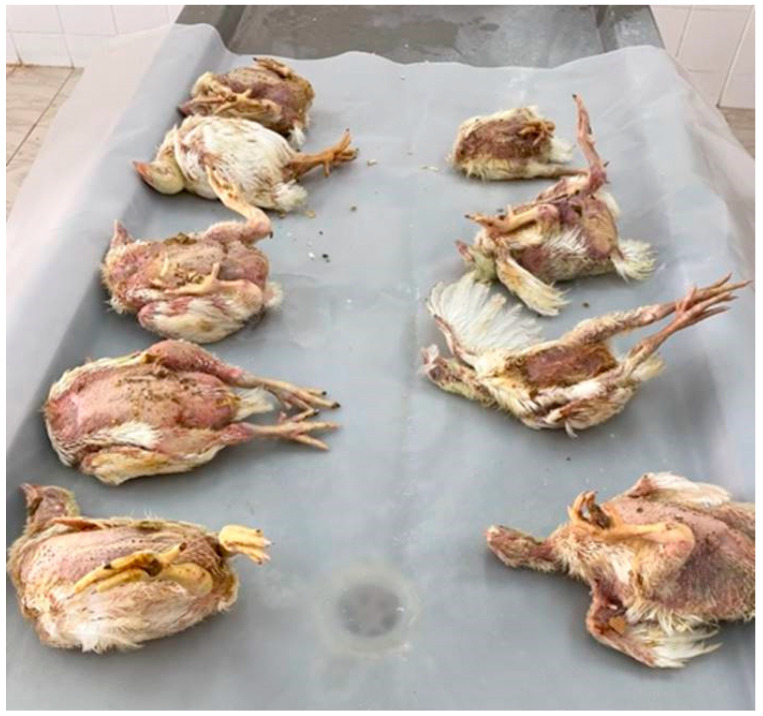
Broiler chicken carcasses infected with FAdV presented for necropsy examination showing rough feather, distended abdomen and diarrhea.

**Figure 3:**
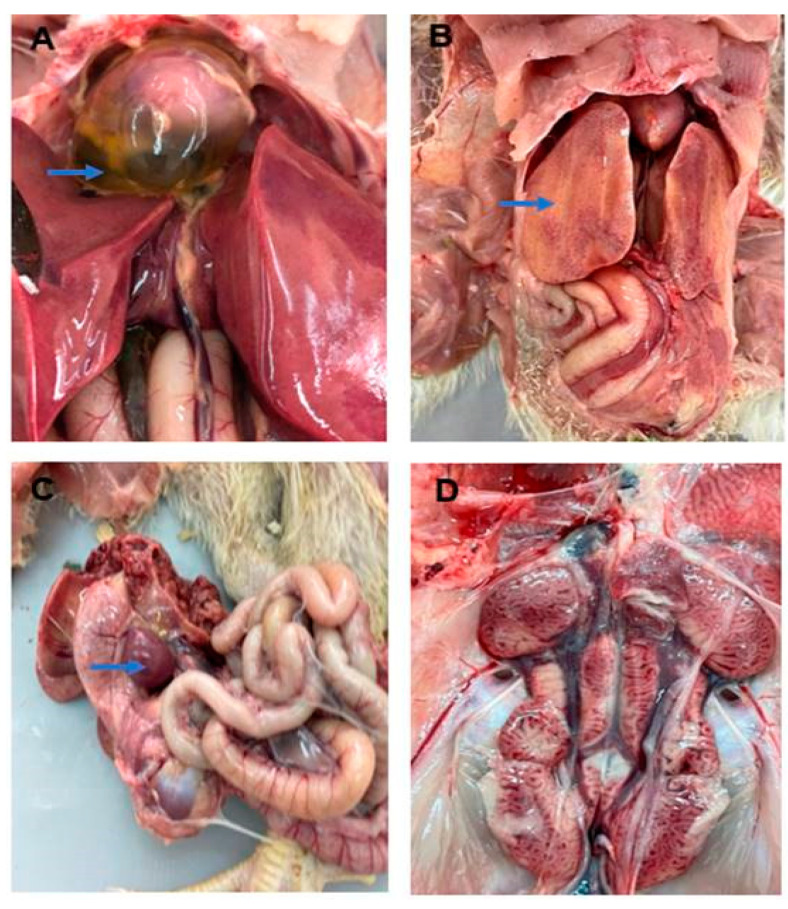
(**A**) The heart—accumulation of clear, straw-colored fluid in the pericardial sac (blue arrows). (**B**) The liver—pale, necrotic enlarged and friable (blue arrow). (**C**) Spleen—enlargement, congested and mottled spleen. (**D**) Kidneys—enlargement, congested, pale prominent tubules.

Affected flocks showed various signs of sudden onset, high morbidity and mortality rates, rough feather, diarrhea, general weakness and finally, deaths (Figure 2). Necropsy examination revealed prominent gross lesions including pale, enlarged, hemorrhagic, necrotic and friable liver; hydropericarditis with the accumulation of straw-colored fluids in the pericardial sac; pale kidneys with enlarged prominent tubules; and mottled splenomegaly (Figure 3).

### 3.3. Histological Analysis

**Figure 4:**
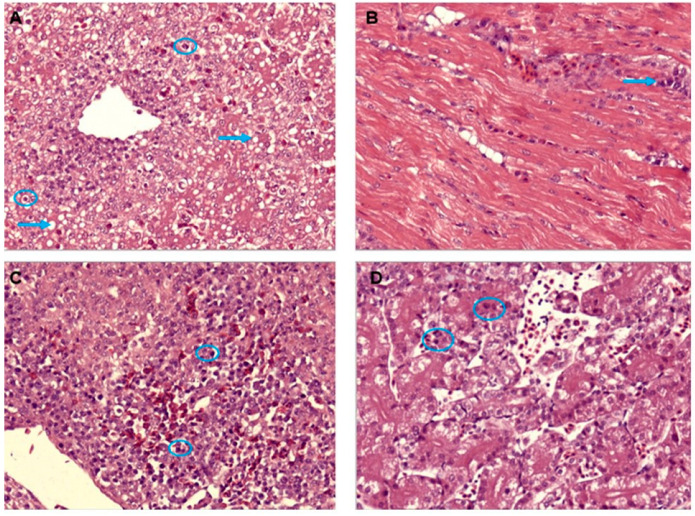
Histologic lesions of natural Avian Adenovirus infection. H&E staining, ×400. (**A**) The liver, hydropic and vacuolar degeneration in hepatocytes (blue arrows) with basophilic intranuclear inclusion body in liver cells (blue circles). (**B**) The heart—monocytes and macrophages infiltration in interstitial areas (blue arrow). (**C**) The spleen—reticuloendothelial cells hyperplasia with intranuclear inclusions bodies (blue circles). (**D**) Kidney—hyperemia and degeneration of the epithelium in renal tubules with intranuclear inclusions bodies (blue circles).

The most prominent microscopic lesion in the liver was multifocal hepatitis with accumulation of mononuclear cells in some areas, hydropic and vacuolar degeneration in hepatocytes, and many hepatocytes had large, round, eosinophilic or basophilic intranuclear inclusion bodies (Figure 4A). There was an accumulation of mononuclear cells and macrophages in the heart in some interstitial areas (Figure 4B). In the spleen, reticuloendothelial cells hyperplasia with intranuclear inclusions bodies was observed (Figure 4C). In the kidneys, the lesions were variable. Some birds showed severe hyperemia and massive degeneration of renal epithelial cells, while other birds demonstrated edema fluid and intranuclear inclusions bodies (Figure 4D).

### 3.4. Molecular Analysis

#### 3.4.1. The qPCR

**Figure 5:**
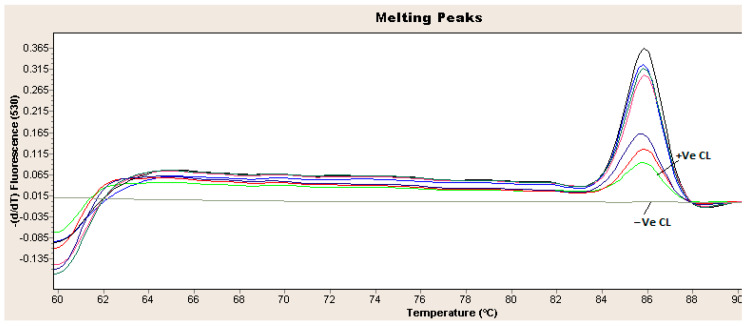
Real-time PCR amplification curves. Melting curve of the amplified FAdV-4 hexon gene from infected chickens tested tissue specimens (heart, spleen, kidney or liver). The arrows indicate positive control (+Ve CL) and negative control (−Ve CL). The qPCR was performed with LightCycler^®^ 2.0 Instrument.

Out of ninety-seven necropsied chickens, only fifty-four tissue samples from all farms, including liver, spleen, heart and kidney, were collected and subjected to qPCR analysis. All the samples from farms were tested positive for FAdV (A–E) by qPCR (100%). The Ct values of the samples ranged between 15 and 25. The melting curve of the qPCR using SYBER Green chemistry is shown in Figure 5.

#### 3.4.2. Sequencing and Phylogenetic Analysis of FAdV

**Figure 6:**
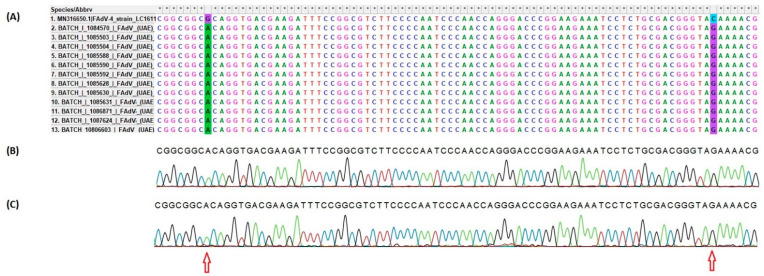
Mapping of the hexon gene sequence of FAdV of UAE field strains to the reference genome of FAdV serotype 4 strain LC1611 hexon gene (Sequence ID: MN316650.1) (**A**), while (**B**,**C**) represents the sequence and the chromatogram of two FAdVs hexon genes from UAE field strains, farm 1 and farm 2, respectively. The mutations G > A and C > G were highlighted in green and blue, respectively, while red arrows denoted their chromatogram. * denotes for 100% identity.

Serotyping of FAdVs field strains was performed by sequencing and phylogenetic analyses of the partial Hexon gene from twelve FAdV field samples (one sample from farms numbered 2, 6 and 7, two samples from farms numbered 1, 3, 5 and three samples from farm number 4). BLAST analysis of the partial hexon gene obtained in this study indicated that the sequence had shared a 99.70% to 99.85% nucleotide similarity with the FAdV serotype 4, species C reference sequences available in the NCBI database with notable two substitution mutations in all UAE FAdV hexon gene sequences as G > A and C > G (Figure 6) without amino acid (AA) deletions. The later mutation changed the amino acid glutamine (Gln or Q) to glutamic acid (Glu or E) (Figure S1). By computing the pairwise distance of partial-length hexon coding sequence of species FAdV-C obtained in this study, it was found that the sequence had a 100% similarity among them and varying between 0.45 and 1% from the hexon gene sequence of FAdV serotype 4, species C of China, Nepal, Pakistan and Saudi Arabia.

**Figure 7:**
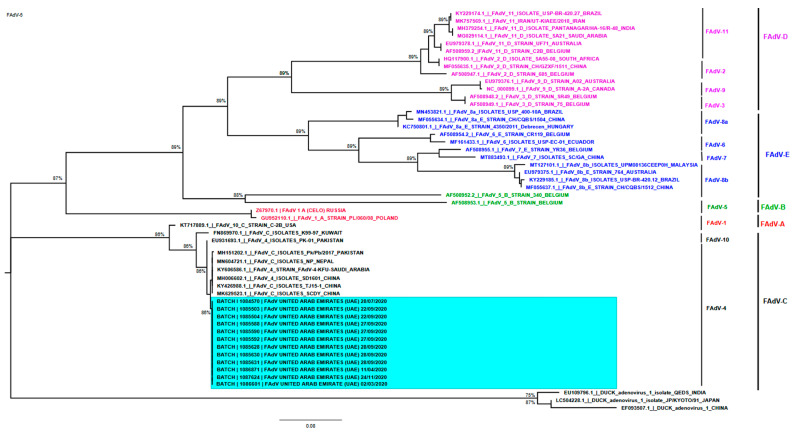
Hexon gene-based phylogenetic analysis of FAdV serotype 4. The tree was constructed using MEGA X software by the Maximum Likelihood method (1000 replicates for bootstrap utilizing the partial FAdV serotype 4 hexon gene obtained from sequencing of twelve FAdV field strains from UAE strain and other forty-one representatives FAdV reference strains obtained from GenBank. The UAE FAdVs are clustered into FAdV serotype 4, species C, with other isolates from Saudi Arabia, Pakistan, Nepal and China. The FAdV serotype 4, species C of UAE field strains, was highlighted. Serotypes are indicated on the right.

The sequence data of 12 field samples obtained from this study were deposited in GenBank under the accession numbers OL456285–OL456296. Phylogenetic analysis indicated all obtained sequences are classified as FAdV group C, serotype 4 as a dominant serotype associated with IBH and HBP in the region (Figure 7). It was noted the UAE FAdV strains clustered along with the same serotype of FAdVs circulating in Saudi Arabia (KY606586.1), Pakistan (MH151202.1 and EU931693.1), Nepal (MN604721.1) and China (MK629523.1, KY426988.1 and MH006602.1) (Figure 7), but forming a unique monophyletic group.

## 4. Discussion

The FAdV infections are associated with several diseases, including IBH, HPS and AGE. In 2020 in the Al Ain region, UAE, outbreaks of IBH and HPS were reported through the routine diagnosis by ADAFSA veterinary laboratories with high morbidity and mortality rates in several broiler farms, leading to severe economic losses. In order to better prevent and control the FAdV infection in the regions, we characterized the UAE FAdV field strains and identified their serotypes based on the sequence of the hexon gene, which could help in the design of an effective vaccination strategy. To the best of our knowledge, this is the first report on FAdV epidemiology in poultry in the region.

In addition to FAdV infection that caused high mortality rates observed (reached 100% in some farms on the date of notification), co-infection with other immunosuppressive viruses such as bursal disease virus (IBDV) and chicken infectious anemia virus (CIAV) could not be excluded as these diseases were also found to contribute to the high IBH and HPS related deaths [19,35]. However, screening of these viruses during FAdV testing was not performed, which is a limitation to the study. Therefore, it is unclear whether FAdV described in this study is a primary or secondary infection. Moreover, it is necessary to evaluate the biosecurity level in these farms as biosecurity is also considered one of the factors contributing to the high incidence of FAdV infections [26].

The IBH and HPS were known to infect chickens of ages 3–5 weeks [36]; similar results were observed in our study as most affected ages are 2–4 weeks, which may indicate both horizontal and vertical transmission. However, it is unclear whether the route of transmission was horizontal or vertical in each case in our study.

Although hepatitis was a prominent finding, the viral inclusion bodies were regularly observed in the present study. Necropsy examination of the affected chicken revealed pale, enlarged, hemorrhagic, necrotic and friable liver; accumulation of straw-colored fluids in the pericardium sac; pale kidneys with enlarged prominent tubules; and enlarged mottled spleen. A similar clinical picture was reported in chickens in many FAdV serotype 4 outbreaks in several countries, including India, Pakistan, Iraq and Korea [6,23,37,38].

The PCR investigation has confirmed that FAdV was the causative agent of IBH and HPS in broiler chickens in UAE in 2020. We monitored the presence of FAdV serotype 4 DNA in tissue samples collected from necropsied chickens using previously published primers [20]. The results further described the liver; hearts, spleen and kidneys are the most affected organs in chickens infected with FAdV serotype 4. These results match well with the typical clinical picture of FAdV serotype 4 in affected birds that have been reported elsewhere [15,39].

The FAdV infection in Pigeon in Dubai, UAE, was previously reported [27], but the serotype and species were not described. In contrast, in our study, we found that all the FAdV field strains affected broiler chickens were genetically related to serotype 4, species C. All the twelve partial hexon genes sequenced from UAE FAdV serotype 4 field strains obtained from different outbreaks are 100% identical, which suggests a single strain circulating in these different farms and may derive from a common ancestor or common source, although the epidemiological relationship is unclear. These UAE strains were also found to have two mutations in hexon gene G > A and C > G (Figure 5) without amino acid (AA) deletions, but the later mutation changes the amino acid from glutamine (Gln or Q) to glutamic acid (Glu or E). Structurally, glutamine has a side chain similar to that of glutamic acid, except the carboxylic acid group is replaced by an amide group. The glutamic acid serves as the precursor for synthesizing the inhibitory gamma-aminobutyric acid (GABA) in GABA-ergic neurons. Interestingly, the hexon protein and the fiber proteins were described to play a major role in FAdV serotype 4 pathogenicity [40]. Specifically, the amino acid residue at position 188 of the hexon is responsible for FAdV serotype 4 pathogenicity [41]. However, the contribution of the amino acid substitution observed in this study to the pathogenicity of FAdV serotype 4 UAE field strains remains to be studied. Thus, it is necessary to continue investigating the prevalence of FAdV and understand the genetic epidemiology of viruses associated with IBH and HHS across the UAE.

Furthermore, sequencing analysis of the partial hexon gene revealed a high similarity of the FAdV serotype 4 strain in this study to those from Asian and Gulf countries such China, KSA, Nepal and Pakistan, which may indicate the circulation of FAdV serotype 4 in these regions and implies the application of further control measures to reduce the FAdV burden. However, the FAdV from this study formed a unique monophyletic group in the phylogenetic tree, which could be explained by the mutations in two regions (G > A and C > G) in UAE FAdV hexon gene sequences, as explained earlier in (Figure 5). By computing the pairwise distance of partial-length of hexon gene sequence obtained in this study, with hexon gene sequence from close related FAdV serotype 4, species C shown in the phylogenetic tree, it was found that the sequence FAdV UAE sequences had a 100% similarity between them and varying between 0.45 and 1% from hexon gene sequence from China, Nepal and Pakistan and Saudi Arabia. This may further explain the monophyletic cluster of UAE FAdVs.

Mixed infections of multiple serotypes were observed in other world regions, such as China [1]. Therefore, it is crucial to continue investigating the presence of mixed FAdV infections, which will represent a big challenge to the prevention and control strategy of the disease in the region. At present, no FAdV commercial vaccine was licensed in UAE; therefore, the detected FAdV were considered natural infections. The latter may underline the necessity of taking adequate control measures against FAdV infections through vaccination and enhancing biosecurity levels in broiler farms.

Overall, the present study demonstrates FAdV circulation (particularly FAdV serotype 4, species C) and the limited effectiveness of applied control measures in preventing the disease occurrence in the region. Currently, only FAdV serotype 4 was associated with clinical FAdV infections, such as IBH or HPS in UAE. Results provided useful information on the genetic epidemiology of FAdV circulating in UAE in 2020 and would help to develop a local vaccine against FAdV serotype 4 serotypes to control and prevent the IHB and HPS diseases among broiler chickens within UAE.

## Figures and Tables

**Table 1:** Epidemiological data of fowl adenovirus infection in chickens in 2020, Abu Dhabi, UAE.

Farm	Outbreak	Age	Sampling Date	Population	Infected	Death	Morbidity Rate (%)	Mortality Rate (%)	Case Fatality Rate (%)
1	1	NA	2 March 2020	NA					
	2	7–14 days	11 April 2020	NA					
2	3	25 days	28 July 2020	9000	9000	6000	100%	67%	67%
3	4	21 days	22 September 2020	20,000	20,000	16,000	100%	80%	80%
4	5	17 days	27 September 2020	16,000	8000	7000	50%	44%	88%
5	6	17 days	28 September 2020	9000	9000	9000	100%	100%	100%
6	7	NA	28 September 2020	107,839	4000	916	3.70%	85%	23%
	8								
7	9	14 days	24 November 2020	11,000	11,000	11,000	100%	100%	100%

## Data Availability

The partial hexon gene sequence generated in this study are available in the NCBI database under accession numbers mentioned in the manuscript.

## Supplementary Materials

The following supporting information can be downloaded at: https://www.mdpi.com/article/10.3390/vetsci9040154/s1, Figure S1: The mutation C > G that changed the amino acid glutamine (Gln or Q) to glutamic acid (Glu or E).
