# Supplementary figures and images for: Pathology and Molecular Epidemiology of Fowl Adenovirus Serotype 4 Outbreaks in Broiler Chicken in Abu Dhabi Emirate, UAE
Source: Vet Sci. 2022 Mar 23;9(4):154. doi: 10.3390/vetsci9040154 (PMC9032256; doi:10.3390/vetsci9040154)

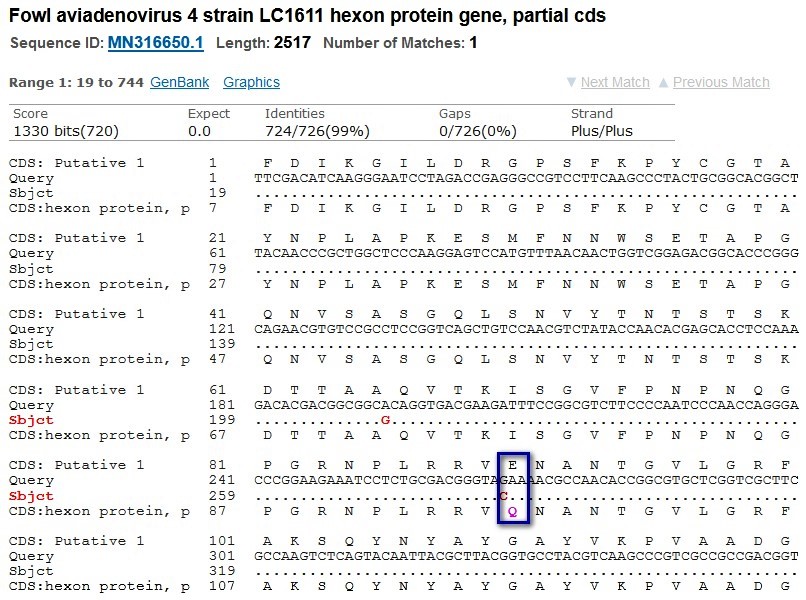

Supplement: Supplementary file 1 [file vetsci-09-00154-s001.zip › vetsci-1613504-supplementary.jpg]
